# Preliminary psychometric performance of the Neonatal Feeding Assessment Scale

**DOI:** 10.4102/sajcd.v64i1.163

**Published:** 2017-01-30

**Authors:** Mari Viviers, Alta Kritzinger, Bart Vinck, Marien Graham

**Affiliations:** 1 Department of Speech-Language Pathology and Audiology, University of Pretoria, South Africa; 2 Department of Statistics, University of Pretoria, South Africa

## Abstract

**Objective:**

The objective was to determine the preliminary psychometric performance of a new clinical feeding scale to diagnose oropharyngeal dysphagia (OPD) in neonates.

**Methods:**

Twenty neonates with a median gestational age of 35 weeks were evaluated using the Neonatal Feeding Assessment Scale (NFAS) and modified barium swallow studies (MBSS). The results were compared.

**Results:**

Nine of the 20 participants presented with OPD on the NFAS. Comparison of the scale’s results with instrumental MBSS indicated that all participants without OPD were correctly excluded (100% sensitivity). The specificity was 78.6%, indicating that three participants were falsely identified with OPD on the scale. Inter-rater reliability was determined on 50% (*n* = 10) of the sample. Substantial agreement (80%) was obtained between two raters in five of the six sections of the scale and on the diagnostic outcome.

**Conclusion:**

The preliminary performance of the scale appears to be promising. A further validation study will take place.

## Introduction

In a developing country such as South Africa there is a need for valid clinical assessment instruments for use by local speech-language therapists (SLTs) in neonatal dysphagia (Viviers, Kritzinger & Vinck, 2016). Such a need was also identified by Botha and Schoeman and indirectly implied in the South African practice guidelines for paediatric dysphagia, as no standardised clinical assessment instrument is recommended to use with neonates (Botha & Schoeman, 2011; SASHLA, 2011a). Due to a lack of regulated service delivery and instrumental assessment equipment available for diagnosing dysphagia in the public healthcare sector, comprehensive clinical assessment may be even more important in developing countries such as South Africa than in developed countries. A limited number of SLTs experienced in the administration and interpretation of modified barium swallow studies (MBSS) or fiberoptic endoscopic evaluations of swallowing are practising in the public and private healthcare sectors. Since objective assessment measures were encouraged there has been a rise in demand for MBSS in the paediatric population, but inadequate radiology infrastructure remains a concern (Hiorns & Ryan, 2006).

Pados, Park, Estrem and Awotwi (2016) found a lack of validated feeding assessment scales for infants younger than 6 months that are supported by high level evidence in a recent review. They concluded that the Early Feeding Skills Assessment Instrument (EFS) was one of the instruments that had some supportive psychometric development and testing in the neonatal population. However, no supportive data on the content validity offered by experts in the area of neonatal feeding for the EFS has been published. Two additional instruments with the most extensive psychometric testing are the Neonatal Oral Motor Assessment Schema (NOMAS) (Palmer, Crawley & Blanco, 1993) and the Schedule for Oral Motor Assessment (SOMA) (Reilly, Skuse & Wolke, 2000), which focus on oral motor skills of the neonate and infant (Pressman, 2010; Rogers & Arvedson, 2005). These two scales do not consider the impact of environmental and internal disruptions on the infant’s physiological subsystems and its resulting effects on the feeding process and mother–infant interaction. In comparison, the EFS aimed to assess oral feeding readiness in a more holistic manner. It is thus recommended that a wider range of infant systems and feeding skills should be evaluated in a comprehensive neonatal clinical assessment instrument than was included in the discussed instruments.

Because neonatal dysphagia services are an important component of early intervention, an assessment instrument should incorporate the principles of family-centred developmentally appropriate care, an asset-based approach, team collaboration and evidence-based practice (ASHA, 2008; Ensher & Clark, 2009; Gooding et al., 2011; SASLHA, 2011b; Thoyre, Shaker & Pridham, 2005). As the parent’s first and enduring caregiving task after birth is to feed the infant, the primary caregiver should be central to the dysphagia assessment process. The value of parental description of the feeding difficulty and observation of a typical feeding routine between the mother and infant during clinical assessment may hold direct benefits for parental compliance during intervention. In contrast, during a MBSS the parent may not be elete central to the assessment procedure.

To respond to the need for a valid neonatal dysphagia assessment instrument for use in resource-constrained developing countries, the Neonatal Feeding Assessment Scale (NFAS) was developed and approved, using expert collaboration through the Delphi method (Viviers et al., 2016). Panel members agreed on a need for a validated NFAS. South African panel members favoured a comprehensive instrument while international members contributed to evidence-based item inclusion and the use of an objective scoring system (Viviers et al., 2016). Clinical assessment will never replace the gold standard of MBSS but may contribute significantly to complex clinical decision-making in neonatal dysphagia. The research question posed for the current study was, ‘what are the preliminary psychometric properties of the newly developed NFAS to diagnose oropharyngeal dysphagia (OPD)?’

## Methods

### Aims and objectives

The aim of the study was to determine the preliminary psychometric performance of the NFAS to diagnose OPD. The objectives were to determine the sensitivity, specificity and accuracy of the NFAS in comparison to the MBSS and to verify inter-rater reliability.

### Design

A comparative within-subject design (Meline, 2010) was used to investigate the psychometric properties of the NFAS by comparing the NFAS and MBSS results.

### Participants

Neonates admitted to a 29-bed neonatal intensive care unit (NICU) at a tertiary academic hospital in Gauteng Province, South Africa, were purposively selected. Mothers were verbally informed of the study and through a brochure in English, Setswana or Afrikaans, the most prominent languages spoken in the city where the study was conducted. Written or verbal (in case of illiterate participants) informed consent was obtained from all mothers. Twenty neonates were selected. The participant inclusion criteria were that the neonate should have a high-risk status such as prematurity, low birth weight (LBW), exposure to HIV or another risk factor (e.g. craniofacial anomaly), predisposing the neonate to feeding and swallowing difficulties; be an in-patient in the NICU; be medically stable for assessment as determined by the treating physician; be within the age range of > 32 weeks gestational age to 4 months corrected age post-term at time of assessment. Neonates younger than 32 weeks gestational age are expected to display feeding and sucking difficulties as a result of immaturity and are typically not fed orally; they were not included. Participant characteristics are presented in Table 1.

**TABLE 1:** Participant characteristics (*n* = 20).

Neonate characteristics	Mean	Median	Mode	SD
Gestational age at birth (duration of pregnancy)	35.15	35.00	32	3.066
Birth weight	2.17	1.94	3.3	0.845
Corrected age at assessment	36.89	36.5	35	2.850
Number of days in NICU	12.65	6.00	6	11.582

SD, standard deviation; NICU, neonatal intensive care unit.

According to Table 1 the participants were born at a mean premature gestational age of 35.15 weeks (SD = 3.066). The mean birth weight of the participants was low, 2.17 kg (SD = 0.845) and the mean length of stay in the NICU was 6 days. Additionally, the sample consisted of slightly more female participants (60%). Other risk factors contributing to feeding difficulties were HIV exposure *in utero* or during delivery (30%, *n* = 6), respiratory distress syndrome (55%, *n* = 11) and hyperbilirubinaemia (55%, *n* = 11). Prematurity (80%, *n* = 16) and LBW (85%, *n* = 17) were the most significant known risk factors for OPD (Pados et al., 2016).

### Materials

The newly developed feeding scale (NFAS) and an MBSS data collection form (based on Arvedson & Brodsky, 2002; Hall, 2001; Swigert, 2010) were used. The MBSS form indicated the stages of swallowing (oral, pharyngeal and oesophageal stages), the presence or absence of any form of dysphagia, and penetration or aspiration in the pharyngeal stage. In addition, a parent interview schedule included pre-, peri- and postnatal information and a description of the feeding problem according to the parents (based on Arvedson & Brodsky, 2002; Hall, 2001; Swigert, 2010). Medical records were used for additional information.

**FIGURE 1:**
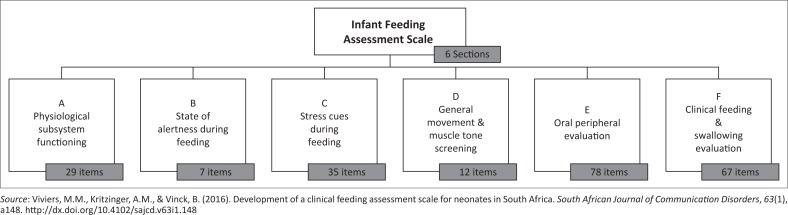
NFAS sections and items.

The development and content of the NFAS were discussed in a previous study (Viviers et al., 2016). The item selection in the sections of the NFAS was based on theoretical constructs related to neonatal and early infant feeding and the clinical assessment of feeding skills. The instrument relies on physiological observations of the infant during feeding, how infant state is influenced by feeding and how feeding may subsequently disrupt a regulated state in the infant with feeding difficulties and an associated display of stress cues (Figure 1).

The MBSS was performed using a fluoroscope (SYSCO 19” version Multi DiagnostEleva FD screening machine from Philips, Amsterdam, Netherlands) with DVD recording capabilities.

### Procedures

Clearance was obtained from the research ethics committee at the university and the medical ethics committee at the tertiary academic hospital where the study was conducted. The mothers of the participants were interviewed, medical files were reviewed, a clinical feeding assessment was conducted using the NFAS and a MBSS was performed. The MBSS was conducted within 7 days of the clinical assessment. The interviews, medical file review and clinical feeding assessments were conducted by the first author, a qualified SLT, and three graduate students in speech-language pathology. All data collectors were trained. Training was provided in a 6-hour session on the content, administration and scoring of the NFAS. After the training session each trainee was expected to accumulate four practice assessments before data collection was initiated. Inter-rater reliability data was obtained for two of the four data collectors (excluding the first author) on 10 infants (50% of sample). Two senior SLTs working at the hospital conducted the MBSS while blinded to the infants’ feeding history and diagnostic outcome of the clinical assessments.

Because feeding is an integrated process, with infant responses in the different sections occurring simultaneously, the order in which sections of the NFAS are completed may vary. A breastfeeding session was observed, or the mother was asked to prepare the bottle feed (expressed breast milk or formula) or supplemented breastfeeding with tube feeding if the infant was not fully breastfed. The complete data collection procedures for the NFAS are presented in Appendix A. Scoring instructions for each section are indicated on the instrument. A binary *yes* or *no* system is used. The outcome of each section is a *yes* or *no* conclusion regarding the possible presence of OPD. Each section score is transferred to the last page of the instrument, where the overall diagnostic outcome of the assessment is calculated. When a score of three or more *yes* responses is obtained, the assessment outcome indicates that OPD is likely to be present. At least one of the three *yes* responses required for reaching the final diagnosis of OPD must either be obtained in Section E or F (Viviers et al., 2016).

During the MBSS a solution of barium sulphate was reconstituted by mixing the powder with the mother’s expressed breast milk or recommended formula. During fluoroscopy the pulsed mode with appropriate collimation was used to limit radiation exposure (Hernanz-Schulman, Goske, Bercha & Strauss, 2011; Scott, Fujii, Behrman & Dillon, 2014). A NUK (Bonn, Germany) MedicPro First Choice^TM^ 120 ml infant bottle with a MedicPro^TM^ disposable thermoplastic elastomer (TPE) Teat Size 1 was used. Participants were positioned with appropriate supported seating in a Tumble Forms 2 Feeder Seat^TM^, Warrenville, United States.

### Data analysis

Frequency distributions were calculated for the NFAS data. Criterion validity was determined by calculating sensitivity (%) and specificity (%) scores based on the comparative data sets. Sensitivity determines the probability of the presence of OPD, whereas specificity reveals the probability that OPD will truly be absent when using the NFAS (Dawson & Trapp, 2004). Positive predictive value (PPV) and negative predictive value (NPV) indicate whether the NFAS predicted the true positive and true negative diagnoses correctly (Dawson & Trapp, 2004). The higher the percentage score derived for PPV and NPV calculations, the better and more valid the predictive ability of the instrument (Dawson & Trapp, 2004). Cohen’s kappa with accompanying asymptotic standard error (ASE) was used to investigate the inter-rater reliability coefficient, together with p-bar calculations for the results obtained by two independent raters. The interpretation of the inter-rater reliability calculations (kappa) according to Dawson and Trapp (2004) and Landis and Koch (1977) are provided in Table 2. A kappa value of greater than 0.41 was considered a minimal reliability criterion (Dawson & Trapp, 2004). Accuracy of agreement between the NFAS and the MBSS diagnosis of OPD was also investigated.

**TABLE 2:** Interpretation guidelines for kappa values for inter-rater reliability.

Kappa values	Interpretation of level of agreement	Kappa values	Interpretation of level of agreement
1.00	Perfect agreement	> 0.75	Excellent agreement beyond chance
0.93–0.99	Excellent agreement		
0.81–0.92	Very good agreement	0.40–0.75	Good agreement beyond chance
It 0.61–0.80	Good agreement		
0.41–0.60	Fair/substantial agreement	< 0.40	Poor agreement beyond chance
0.21–0.40	Slight agreement		
0.01–1.20	Poor/chance agreement		
≤ 0	No agreement	-	-

*Source*: Dawson, B., & Trapp, R.G. (2004). *Basic and clinical biostatistics.* (4th edn.). New York: Lange Medical Books; Landis, J.R., & Koch, G.G. (1977). The measurement of observer agreement for categorical data. *Biometrics, 33*, 159–174. http://dx.doi.org/10.2307/2529310

## Results

### Neonatal Feeding Assessment Scale results

The NFAS was administered on a sample of 20 participants to determine preliminary psychometric properties. The clinical assessment results were compared to the MBSS results to determine which participants presented with true OPD. Table 3 presents the data obtained from the NFAS assessment.

**TABLE 3:** NFAS results (*n* = 20).

Section	Number of infants with indicators for OPD	Frequency distribution (%)
A. Functioning of physiological subsystems?	2	10
B. State of alertness during feeding†	-	-
C. Stress cues during feeding	15	75
D. Movement and muscle tone screening	4	20
E. Oral peripheral examination	8	40
F. Clinical feeding and swallowing evaluation	14	70
Diagnosis of OPD	9	45

OPD, oropharyngeal dysphagia.

† Scoring of Sections A and B are combined on the NFAS.

According to Table 3 nine infants (45%) presented with OPD on the NFAS. Positive identification of OPD could be explained by the participant characteristics (see Table 1) and the previously stated associated risk factors in the sample. As per scoring guidelines, the nine participants obtained a minimum score of three *yes* responses in the five sections, with one of the *yes* responses in either Section E or F of the NFAS. In Sections C (stress cues during feeding) and F (clinical feeding and swallowing evaluation) the highest number of indicators were observed in those neonates diagnosed with OPD. Some of the neonates were not attached to heart rate and respiratory monitors. Therefore certain items could not be scored in Sections A and B (physiological status and state of alertness), resulting in low scores in the combined section. As a result of the low scores in the physiological status and state of alertness sections, the contributions of these sections to diagnose OPD should be investigated further in a larger sample. The NFAS results were then compared to the MBSS results to determine validity.

### Criterion validity

Criterion validity determined the extent to which the NFAS agreed with the gold standard (MBSS) measuring the same variable. Measures to determine criterion validity included the predictive ability, sensitivity, specificity and accuracy of the instrument. The comparative results are presented in Table 4.

**TABLE 4:** Comparison between the MBSS and NFAS results (*n* = 20).

Variable	Results	Outcome of MBSS (*n* = 20)		NFAS: total participants presenting with OPD
		OPD present	OPD absent	
Outcome of NFAS(*n* = 20)	-	True positive	False positive	-
	OPD present	6	3	9
	% NFAS	66.7%	33.3%	100%
	% MBSS	100%	21.4%	-
	-	False negative	True negative	-
	OPD absent	0	11	11
	% NFAS	0%	100%	100%
	% MBSS	0%	78.6%	-
MBSS: total participants presenting with OPD	Count	6	14	20
	% NFAS	30%	70%	100%
	% MBSS	100%	100%	100%

OPD, oropharyngeal dysphagia; MBSS, modified barium swallow studies; NFAS, Neonatal Feeding Assessment Scale.

### Sensitivity and specificity

When comparing the MBSS and NFAS outcomes in Table 4, all six neonates who presented with OPD were correctly identified with the NFAS; however, three were incorrectly identified, resulting in a false positive rate of 21.4%. This comparison revealed the NFAS presented with a sensitivity of 100% when identifying OPD in neonates. The specificity of 78.6% reflects the probability of the NFAS to determine that a neonate does not present with dysphagia.

### Predictive diagnostic ability of the Neonatal Feeding Assessment Scale

The PPV and NPV were calculated using the data in Table 4. The PPV was 100% (6 / 6 × 100) and the NPV was 78.6% (11 / 14 × 100). The higher the PPV and NPV (closer to 100%), the better the new assessment scale is at diagnosing OPD when compared to the gold standard (Parikh, Mathai, Parikh, Chandra Sekhar & Thomas, 2008). Based on the PPV and NPV scores the NFAS showed adequate predictive ability to determine when OPD would be present or absent. It was concluded that among those participants who had OPD the predictive ability of dysphagia being present was 100% and among those participants who did not have OPD the predictive ability of not having dysphagia was 78.6%.

### Diagnostic accuracy of Neonatal Feeding Assessment Scale compared to modified barium swallow studies

The overall accuracy was calculated using the specificity and sensitivity data. The accuracy of agreement on diagnosis of OPD between the NFAS and MBSS was 85% (11 + 6/20 × 100). The closer the accuracy score is to 100%, the better agreement there is between the newly developed instrument and the gold standard (Dawson & Trapp, 2004).

The NFAS therefore presented with good preliminary sensitivity (100%) (Dawson & Trapp, 2004). Specificity was also considered to be good (Dawson & Trapp, 2004) at 78.6%. An assessment tool with a high specificity, sensitivity, PPV, NPV and accuracy is considered valuable in clinical practice (Lalkhen & McCluskey, 2008). The participants not diagnosed with OPD on MBSS presented with oesophageal dysphagia or normal swallowing ability.

Apart from the six participants diagnosed with OPD on the NFAS and the MBSS, the MBSS revealed additional results as expected. Based on MBSS results, 40% (*n*= 8) of the participants presented with oesophageal dysphagia, 10% (*n* = 2) had OPD co-occurring with oesophageal dysphagia and four participants had normal swallowing. Two of the six neonates diagnosed with OPD on the NFAS presented with this co-occurrence.

### Inter-rater reliability

Inter-rater reliability for all the sections and diagnostic outcome of the NFAS between two independent raters were determined using half of the sample (*n* = 10). A kappa value of greater than 0.410 was considered a minimal reliability criterion and a p-bar value of 0.50 (Dawson & Trapp, 2004). The inter-rater reliability calculations of each section of the instrument are presented in Table 5.

**TABLE 5:** Inter-rater reliability of sub-sections and diagnostic outcome of the NFAS (*n* = 10).

Section of NFAS	Kappa	Level of agreement	P-bar	Overall agreement between raters (%)	ASE
A and B	1.000	Perfect agreement	0.90	90% substantial beyond chance	Not applicable
C	0.286	Slight agreement – minimal acceptable level	0.60	60% slight agreement	0.194
D	1.000	Perfect agreement	1.00	100% perfect agreement	N/A
E	0.737	Substantial beyond chance	0.90	90% substantial beyond chance	0.241
F	0.615	Substantial agreement	0.80	80% substantial agreement	0.225
Agreement on NFAS outcome	0.737	Substantial beyond chance	0.90	90% substantial beyond chance	0.241

ASE, asymptotic standard error; NFAS, Neonatal Feeding Assessment Scale.

The inter-rater reliability for two of the five sections of the instrument demonstrated substantial agreement beyond chance. In the combined Sections A and B as well as for Section D, the assessment criteria were clear (0.90–1.00 p-bar), therefore rendering the kappa calculation obsolete for these sections. For Section C the results indicated only slight agreement, which may be due to the variability of infant state during the feeding process. Thus the variability inherent to infant state may have increased the difficulty to evaluate this section objectively. The two raters agreed on the instrument outcome in 90% (*n* = 9) of the cases. The agreement on diagnostic outcome between the two raters was considered substantial beyond chance with an ASE of 0.241 (Dawson & Trapp, 2004).

## Discussion

The preliminary performance of the NFAS indicated that it is a valid method of assessing neonatal feeding skills, guiding clinicians to diagnose OPD and thereby potentially facilitating early detection and management of OPD. According to DeMauro and colleagues, dysphagia is a significant disorder in preterm infants in developing countries, and valid assessment instruments can compensate for the lack of population-based studies (DeMauro, Patel, Medoff-Cooper, Posenscheg & Abbasi, 2011). The prevalence of OPD (45%) found in this sample was higher than in some other studies (DeMauro et al., 2011). In 2014, Zehetgruber and colleagues reported a prevalence range of dysphagia in their sample of preterm and LBW infants of 25%–35% (Zehetgruber et al., 2014). The higher prevalence rate in this study may not be accurate because prevalence cannot be determined on such a small sample as utilised in this study. The NFAS provides more descriptive information on feeding skills, such as detailed information on stress cues and infant state, than the MBSS. Therefore it may also offer more intervention guidelines to inexperienced clinicians.

### Criterion validity

The high sensitivity and specificity of the NFAS provide evidence of the ability of the scale to accurately diagnose the presence of OPD and in turn to also recognise the absence of OPD, rendering very few false positives. There appears to be limited information on the sensitivity and specificity properties of comparable assessments for oral motor difficulties in neonates and infants, such as the EFS, NOMAS and SOMA (Da Costa, Van Den Engel-Hoek & Bos, 2008). The diagnostic accuracy (85%) of the NFAS and its good predictive ability (Dawson & Trapp, 2004) in clinical use showed that the scale is capable of measuring what it intends to measure.

As expected of a direct instrumental observational procedure, the MBSS gave additional diagnoses. Different types of dysphagia exist in neonates, depending on the stage of swallowing that is affected (Pados et al., 2016). Different types of dysphagia can also co-occur. The MBSS diagnosed oesophageal dysphagia and clearly showed the co-occurrence of the two types of dysphagia, OPD and oesophageal dysphagia. Because the focus of SLTs is on assessment and intervention of OPD, preliminary results indicate that the NFAS could serve this purpose. All participants who truly presented with OPD were identified. When relying on clinical assessments only in contexts where MBSS is not available, the three false positive OPD results may not be viewed as disadvantageous. Further research is required to determine whether subsequent assessments on the same neonate using the NFAS may show different results.

## Inter-rater reliability

The preliminary testing of the NFAS showed that acceptable inter-rater reliability was present. Due to the substantial agreement beyond chance achieved in the inter-rater reliability results, it appears that more than one clinician is likely to obtain the same results when using the NFAS. The pre-assessment training and test administration guidelines may be sufficient to support a clinician to obtain consistent results when administering the scale. The NFAS compares favourably with other widely used instruments investigating components of feeding skills, such as the NOMAS (Palmer et al., 1993) and the SOMA (Reilly et al., 2000), which presented with good inter-rater reliability for clinical use in neonates and infants older than 8 months, respectively. A 2008 study by Da Costa and Van der Schans determined the inter-rater reliability of the NOMAS ranged from moderate to substantial agreement (kappa: 0.40–0.65) (Da Costa & Van der Schans, 2008), although Palmer et al. (1993), the developers of the scale, did not test the final scale for reliability. The SOMA presented with a kappa of <0.75 on a sample of 10 infants, indicating excellent agreement beyond chance (Reilly, Skuse, Mathisen & Wolke, 1995). The authors of the EFS (Pados et al., 2016) stated that intra- and inter-rater reliability had been found to be stable and acceptable, but no data were provided to support this statement (Da Costa & Van der Schans, 2008). The preliminary results thus indicate good reliability (Dawson & Trapp, 2004) of the NFAS.

## Scoring criteria

The weighting of the different sections of the NFAS, in contributing to the diagnosis of OPD, could not be determined adequately in this study due to the small sample size. It appears that state observation (Section B) may be difficult to score due to the fleeting nature of infant states and fluidity between some state changes during a feeding session. Simultaneous observation of different feeding skills in the infant is required when using the NFAS. While focusing on the oral area to observe aspects such as non-nutritive sucking (NNS) and the neonate’s behavioural response to NNS during feeding, there may also be subtle stress cues and state changes taking place, with the result that some of the state changes and stress cues may be missed. In premature and LBW infants, state is influenced by a variety of factors, such as energy expenditure and endurance during feeding (Arvedson & Brodsky, 2002; Thoyre, Park, Pados & Hubbard, 2013). Nugent, Keefer, Minear, Johnson and Blanchard (2007) concurred that the accuracy of state observation requires that clinicians gain clinical experience and attend continued professional development training opportunities in the observation and interpretation of neonatal and infant behaviour. Because state regulation not only impacts on feeding but on the full spectrum of infant behaviour, it may not directly contribute to the diagnostic process during feeding assessment. Observation of state regulation is, however, recognised in the literature and other studies (Browne & Ross, 2011; Nugent et al., 2007). Evaluation of state regulation may help the clinician to support the parent to identify infant states and understand that certain activities are more appropriate while the infant is in one particular state than another. For example, feeding is best supported when an infant is in one of the alert states (Stage 4, quiet alert) without showing distress (Browne & Ross, 2011; Nugent et al., 2007). Feeding in itself also acts as the initial primary regulator of physiological state, as the very young infant uses primitive brainstem–visceral circuits during feeding as the underlying mechanism for state regulation (Browne & Ross, 2011).

## Conclusion

In summary, neonatal dysphagia will remain a complex problem that requires multidisciplinary, multidimensional assessment and treatment. In order to increase effective management of neonatal feeding and swallowing difficulties, the standard of clinical assessment should improve in developing countries where services are not well regulated. The use of validated neonatal feeding assessment instruments should take priority to support evidence-based practice (Miller, 2009; Pados et al., 2016).

A comprehensive clinical assessment instrument addressing the overall feeding process in neonates that also provides systematic guidance in clinical decision-making for the diagnosis of OPD is recommended. The NFAS highlights the subtleties of the feeding process and describes procedures of observation and elicitation that should not be overlooked during clinical assessment. Multidisciplinary team members and newly qualified or inexperienced clinicians should be able to use such an instrument if sufficiently prompted by the systematic procedures for administration outlined in the tool.

The different sections and items in the NFAS may assist to describe the feeding profile of high-risk neonates and consequently enable early and accurate clinical diagnosis of OPD in the absence of available instrumental assessments in resource-constrained contexts. The validity of an assessment instrument is its real capacity to measure what it proposes to measure. This preliminary attempt at validation of the NFAS was performed by comparing it to the MBSS. A larger sample will be utilised to determine psychometric properties of the NFAS for clinical use in a follow-up study. In addition, the contribution of the different sections of the NFAS to the eventual diagnosis of OPD in a neonate will also be investigated.

## Appendix 1

**TABLE 1-A1:** Data collection procedures for the NFAS.

Sections of the NFAS	Procedures
A: Physiological subsystem functioning	Since respiratory problems are one of the most common causes of paediatric dysphagia, assessment of respiratory patterns during feeding was included. Respiratory rate and heart rate may further reveal signs of dysphagia and possible chronic aspiration. The data collectors observed heart rate and respiratory rate as well as the presence of abnormal respiratory patterns.
B: State of alertness during feeding	As infant state typically varies during feeding, behaviour should be assessed to determine the optimal stage of alertness to proceed with oral feeding. The infant should be in an optimal state of alertness for successful oral feeding. The different stages of alertness and subsequent impact on feeding ability were informed by the Synactive Theory of Development. The neonate’s state of alertness during feeding was observed and documented by the data collectors.
C: Stress cues during feeding	An infant’s ability to respond to incoming sensory information plays a role in feeding readiness. The interaction between the state regulation, motor system and autonomic nervous system should be observed, to determine stress during feeding and to enable the clinician or parent to make adaptations. The data collectors observed and documented all stress cues present during feeding.
D: General movement and muscle tone screening	Adequate postural control is a prerequisite for safe and efficient feeding. Inadequate muscle tone, postural control or movement may impact negatively on oral feeding. The data collectors observed and documented postural control, muscle tone and movement patterns at rest and during feeding.
E: Oral peripheral evaluation	Successful swallowing requires the coordination of 31 muscles and five cranial nerves. Infant anatomy, physiology, primitive oral reflexes and underlying cranial nerve function were assessed at rest and during feeding and then results were documented.
F: Clinical feeding and swallowing evaluation	The purpose of clinical assessment is to observe the oral preparatory/oral stage of swallowing and make certain inferences about the pharyngeal stage, provide baseline feeding and swallowing data for further management, and determine progress. The data collectors observed feeding and swallowing during a habitual feeding session with the mother. The data collector elicited NNS response during evaluation of oral primitive reflexes in the previous section, observed NS skills, observed whether there were any signs of avoidance behaviour during NS, and observed and documented signs and symptoms of oral stage difficulties and suspected pharyngeal stage difficulties.

NS, nutritive sucking.
